# Translocator Protein 18 kDa (TSPO) Deficiency Inhibits Microglial Activation and Impairs Mitochondrial Function

**DOI:** 10.3389/fphar.2020.00986

**Published:** 2020-06-30

**Authors:** Rumeng Yao, Ruiyuan Pan, Chao Shang, Xiaoheng Li, Jinbo Cheng, Jiangping Xu, Yunfeng Li

**Affiliations:** ^1^ Department of Neuropharmacology and Drug Discovery, School of Pharmaceutical Sciences, Southern Medical University, Guangzhou, China; ^2^ State Key Laboratory of Toxicology and Medical Countermeasures, Beijing Key Laboratory of Neuropsychopharmacology, Beijing Institute of Pharmacology and Toxicology, Beijing, China; ^3^ The State Key Laboratory of Brain and Cognitive Sciences, Institute of Biophysics, Chinese Academy of Sciences, Beijing, China; ^4^ Institute of Military Veterinary Medicine, Academy of Military Medical Science, Changchun, China; ^5^ The Brain Science Center, Beijing Institute of Basic Medical Sciences, Beijing, China; ^6^ Key Laboratory of Modern Preparation of TCM, Ministry of Education, Jiangxi University of Traditional Chinese Medicine, Nanchang, China; ^7^ Center on Translational Neuroscience, College of Life & Environmental Science, Minzu University of China, Beijing, China; ^8^ Central Laboratory, Southern Medical University, Guangzhou, China

**Keywords:** TSPO, microglia, neuroinflammation, phagocytosis, mitochondrial, metabolism

## Abstract

TSPO is mainly expressed in the mitochondrial outer membrane of microglia in the central nervous system, and its expression is greatly increased when microglia are activated. However, the role and mechanism of this protein in microglial activation is not well characterized. In this study, we investigated the role of TSPO in microglial activation by isolating primary microglia from *TSPO* knockout mice and constructing TSPO-knockdown microglial cell line. We found that TSPO deficiency significantly inhibited microglial activation induced by LPS or IL-4. Mechanistically, TSPO deficiency greatly decreased the mitochondrial membrane potential and ATP production. Moreover, an analysis of cellular energy metabolism showed that TSPO deficiency suppressed mitochondrial oxidative phosphorylation (OXPHOS) and glycolysis, resulting in microglial overall metabolic deficits. Together, our results reveal a crucial role of TSPO in microglial activation through the regulation of mitochondrial metabolism, thus providing a potential therapeutic target for neuroinflammation-related diseases of the central nervous system.

## Introduction

Translocator protein 18 kDa (TSPO), also known as peripheral benzodiazepine receptor (PBR), is primarily localized in the outer mitochondrial membrane and binds with voltage-dependent anion channel (VDAC) and the adenine nucleotide transporter (ANT) (McEnery et al., 1992; Papadopoulos et al., 2006). TSPO is mainly distributed in steroidogenic tissues, and one important function is translocating cholesterol from the cytoplasm into mitochondria, which is the rate-limiting step in the synthesis of neurosteroids and other steroids (Papadopoulos et al., 2006; Papadopoulos et al., 2007). It is worth noting that TSPO is also expressed in the central nervous system (CNS) (Lacapere and Papadopoulos, 2003), with high levels in microglia and low levels in neurons and astrocytes (Kuhlmann and Guilarte, 2000; Maeda et al., 2007; Cosenza-Nashat et al., 2009). However, when inflammation and injury occur in the brain, TSPO expression is markedly increased in microglia and astrocytes, suggesting that TSPO may play an important role in the activation of microglia and astrocytes (Kuhlmann and Guilarte, 2000; Le Goascogne et al., 2000). Furthermore, clinical imaging studies also indicate that TSPO significantly increases in neurodegenerative and neurological diseases such as Alzheimer’s disease (AD) and Parkinson’s disease (PD) (Ouchi et al., 2005; Gerhard et al., 2006; Edison et al., 2008; Yasuno et al., 2008). Hence, TSPO has been proposed as a biomarker of neuroinflammation, and TSPO ligands are being developed as neuroimaging agents (Rupprecht et al., 2010).

Microglia, the resident immune cells of the CNS, play a critical role in neuroinflammation (Aguzzi et al., 2013; Salter and Stevens, 2017), which is closely associated with the occurrence and development of various brain diseases (Stone et al., 2009; Xanthos and Sandkuhler, 2014; Heppner et al., 2015; Heneka et al., 2015). Under different environmental cues, microglia can differentiate into distinct functional types. For example, upon lipopolysaccharide (LPS) or interferon-γ (IFN-γ) treatment, microglia polarize to the classically (M1) activated state, characterized by the production of destructive proinflammatory cytokines (David and Kroner, 2011; Hu et al., 2015; Ji et al., 2019). In contrast, interleukin-4 (IL-4) could drive microglia into the alternatively (M2) activated state, characterized by high phagocytosis activity and the release of some anti-inflammatory factors (David and Kroner, 2011; Hu et al., 2015; Ji et al., 2019). Recently, an increasing number of studies have suggested that microglial polarization is closely related to cellular metabolism (Ulland et al., 2017). Insufficient energy supply results in microglia phagocytosis dysfunction, which accelerates the development of neurodegenerative diseases (Engl and Attwell, 2015; Kalsbeek et al., 2016).

Multiple pieces of evidence show that TSPO ligands, including PK11195, AC-5216, Ro5-4864, and so on, are involved in the regulation of microglial M1 activation (Choi et al., 2011; Karlstetter et al., 2014; Azrad et al., 2019). However, as each TSPO ligand has different binding sites that cause different specificity and selectivity (Bader et al., 2019), there is still no clarification of the specific role of TSPO in microglial activation of M1. In addition, whether TSPO is involved in microglial M2 state activation is still not clear. All of these questions still need to be explored.

In the present study, we found that TSPO deficiency significantly inhibited microglial activation and decreased mitochondrial membrane potential and ATP production. Furthermore, knockout of *TSPO* suppressed mitochondrial oxidative phosphorylation (OXPHOS) and glycolysis. Taken together, our results demonstrate that TSPO plays a vital role in microglial activation, implicating TSPO as a potential therapeutic target in CNS diseases.

## Materials and Methods

### Animals

*TSPO* heterozygous (HZ, +/-) mice on the C57BL/6 background were generated by the Cre-*LoxP* System, which was provided by Professor Jian-Min Zhang of the Chinese Academy of Medical Science. TSPO^−/−^ mice were bred with TSPO^+/-^ mice. Confirmation of genotypes by PCR analysis of tail biopsy specimens. All experimental animal procedures were approved by the Institutional Animal Care and Use Committee of the Beijing Institute of Basic Medical Sciences.

### Primary Microglial Cell Culture

Primary microglia were generated by postnatal 0 to 3 days in wild-type (WT) or TSPO^−/−^ mice. The procedure was modified from a previous report (Yin et al., 2016). Briefly, brains were dissected from postnatal 0- to 3-day-old mice and then dissociated by 0.25% trypsin and trituration until no small clumps were observed in the cell suspension. Single-cell suspensions were obtained by passing the suspension through a 70-μm nylon cell strainer. Finally, the cell suspension was plated onto 75 cm^2^ poly-L-lysine-coated culture flasks. The cells were grown in Dulbecco’s modified Eagle medium (DMEM, Invitrogen, Waltham, MA, USA) supplemented with 20% fetal bovine serum (FBS, Gibco, Grand Island, NY, USA) and 1% penicillin-streptomycin (Invitrogen). After 10 days of culture, primary microglia were separated and collected for experiments.

### Purity of Microglia Cultures

Immunocytochemistry (ICC) was used to confirm microglia purity. A total of 1×10^5^ microglia per well were plated onto poly-L-ornithine-coated 24-well plates. Anti-Iba1 antibody (NB100-1028SS, Novus Biologicals, Littleton, CO, USA) was used to label microglia, and DAPI (4′,6-diamidino-2-phenylindole) (Invitrogen) was used to visualize the nuclei. Fluorescence images were captured using a Nikon confocal microscope. The percentage of microglia was determined by dividing the number of Iba-1-positive cells by the total number of DAPI-positive cells.

### shRNA Knockdown of TSPO in BV2 Cells

In order to stably knock down TSPO in BV2 cells, shRNA against TSPO (targeting sequence: GTGGTATGCTAGCTTGCAGAA) was used. Briefly, the sequences of shRNA were annealed and ligated into a PLKO.1 lentiviral vector (Addgene, Cambridge, MA, USA) and then cotransfected with viral packaging plasmids (ΔR812 and VSVG) into HEK 293T cells. The supernatants containing the lentiviral particles were harvested at 48 and 72 h after transfection. Then, the supernatant was used to infect the BV2 cells with polybrene (sc-134220, Santa Cruz Biotechnology, 1:1000). Two days after infection, cells were subjected to puromycin selection until no cells died, and knockdown efficiency was detected by Western blot and RT-qPCR.

### Western Blotting

Western blotting analyses were performed as described previously (Wu et al., 2016; Zhou et al., 2017). The following antibodies were used: rabbit monoclonal anti-TSPO (ab109497, Abcam, Cambridge, MA, USA), rabbit monoclonal anti-iNOS (D6B6S, Cell Signaling Technology, Cambridge, MA, USA), mouse monoclonal anti-β-actin (66009-1-Ig, Proteintech, Wuhan, China), mouse monoclonal anti-β-tubulin (CW0098M, CWBiotech, Beijing, China), and rabbit monoclonal anti-arginase-1 (93668S, Cell Signaling Technology).

### Measurement of Gene Expression Changes by qRT-PCR

RNA was isolated from primary microglia and BV2 and then extracted using TRIzol (Invitrogen). Total RNA (1 μg) was used for reverse transcription using a cDNA synthesis kit (AT311-01, TransGen Biotech) according to the manufacturer’s instructions. Quantitative real-time PCR was performed using SYBR green master mix (Bio-Rad) on a Bio-Rad iCycler iQ Real-Time PCR system. β-Actin or GAPDH was used as a housekeeping gene for normalization. The mRNA expression analysis was performed using the Delta-Delta CT method.

### Phagocytosis Assays

BV2 cells were placed onto 12-well plates at a density of 3×10^5^ cells per well in DMEM with 10% FBS. Cells were cultured at 37°C overnight. Latex beads (6 µm, internally dyed with the fluorophore Flash Red; Polysciences, Inc. Warrington, US) were incubated in 50% PBS and FBS. BV2 cell media was replaced with DMEM 12 hours before adding preincubated beads to the cells at a concentration of ten beads per cell. BV2 cells and beads were co-incubated at 37°C for 24 hours. Then, the cells were washed twice with PBS, and the cell suspension was prepared by trypsinization and analyzed by flow cytometry.

### Measurement of Mitochondrial Membrane Potential

Mitochondrial membrane potential (Ψm) was measured using tetramethylrhodamine methyl ester (TMRM; T668, Invitrogen, Waltham, MA, USA). Primary microglia were seeded onto 12-well plates. After overnight incubation, microglia were loaded with 20 nM TMRM for 30 min at 37°C. Then, the cells were washed twice with PBS, and the cell suspension was prepared by trypsinization and analyzed by flow cytometry with a 488-nm laser for excitation and a 570 ± 10 nm emission filter.

### Cell Viability Assay

Cell viability assays were performed using a Cell Counting Kit (CCK) (FC101-02, TransGen Biotech, Beijing, China) according to the manufacturer’s protocol. Briefly, 5×10^3^ cells per well were placed into 96-well plates, 10 µl CCK solution was added at the indicated time, and the plates were incubated for 2 hours in a cell culture incubator. Absorbance was measured at 450 nm using a microplate reader.

### Measurement of Total mtDNA and Cytosolic mtDNA

BV2 cells were plated into 6-well plates at a density of 6×10^5^ per well. Total DNA was extracted using the Allprep DNA/RNA Mini Kit (80204, Qiagen) according to the manufacturer’s protocol. mtDNA was quantified by qPCR as described (Zhong et al., 2018) using a specific region of mtDNA not inserted into nuclear DNA (non-NUMT). Nuclear DNA encoding B2m was used for normalization. The mtDNA in the cytosol was measured as described (Nakahira et al., 2011). Briefly, DNA was isolated from 200 µl of the cytosolic fractions, which had been normalized by cytosolic protein concentrations, and mtDNA levels were quantified by qPCR as described above.

### Measurement of ATP Levels

ATP levels were determined using CellTiter-Glo Reagent (G7573, Promega, Madison, WI, USA) according to the manufacturer’s protocol. In brief, 2 × 10^4^ cells per well were placed onto 96-well plates and cultured overnight. The following day, the supernatant was discarded, and 100 μl lysis buffer containing luciferase reagents was added and incubated for 10 min at room temperature. Fluorescence intensity was determined using a microplate reader, and the data were normalized to WT groups.

### Seahorse Extracellular Flux Assay

The oxygen consumption rate (OCR) and the extracellular acidification rate (ECAR) were performed using the Seahorse XF Cell Mito Stress Test Kit (103015-100, Agilent Technologies) and Seahorse XF Glycolysis Stress Test Kit (103020-100, Agilent Technologies) according to the manufacturer’s protocols. In brief, 3×10^5^ primary microglia per well were seeded onto a Seahorse XF 96-well culture microplate overnight. Upon measurement, cells were washed twice with XF assay medium (102353-100, Agilent Technologies) and maintained in it. After baseline measurements, oligomycin, carbonyl cyanide p-trifluoromethoxyphenylhydrazone (FCCP) and Rotenone (Rote), and antimycin A (AA) were injected into the wells sequentially at specific time points for OCR analysis. For ECAR analysis, glucose, oligomycin, and 2-DG were injected. Seahorse XF 96 Wave software was used to analyze the data. The results were normalized to cell number, and data are presented as pmol/min for OCR and mPH/min for ECR.

### Statistical Analysis

All data are indicated as the mean ± SEM. Statistical analyses were performed using GraphPad Prism version 6.0 software. The significance of differences was calculated using Student’s t-test or one-way or two-way analysis of variance (ANOVA) followed by Tukey’s multiple comparisons test as indicated. For all tests, P<0.05 was considered statistically significant.

## Results

### Deficiency of TSPO Inhibits Microglial M1 Polarization

**Figure 1:**
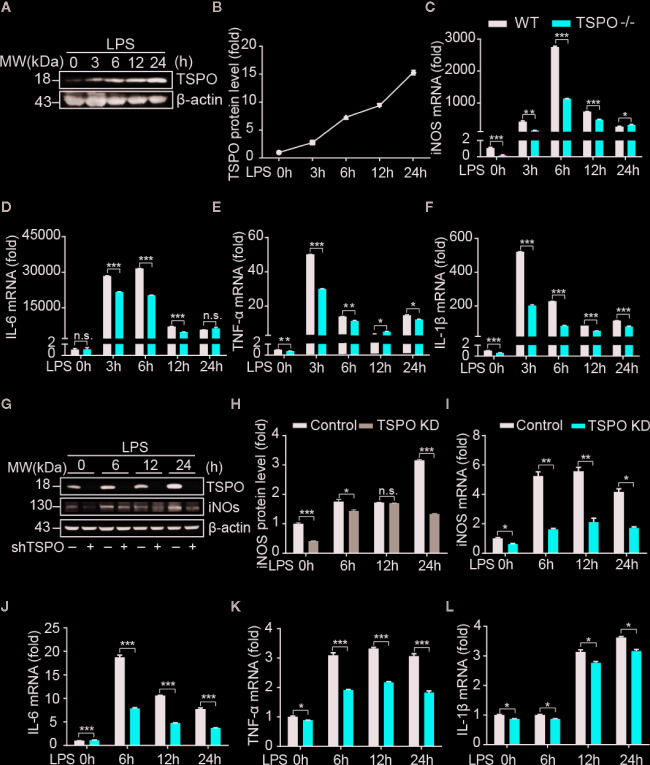
TSPO deficiency inhibits LPS-induced inflammation in microglia. WT and TSPO^−/−^ primary microglial cells were stimulated with LPS (1 µg/ml) for the indicated amounts of time. **(A, B)** Western blot analysis of TSPO protein changes after LPS stimulation. **(C–F)** RT-qPCR determined mRNA changes in the proinflammatory genes iNOS, IL-6, TNF-α, and IL-1β. The same conditions were applied to shvector and shTSPO BV2 cells. **(G, H)** Western blot analysis of TSPO and iNOS levels in control and TSPO-knockdown BV2 cells. **(I–L)** RT-qPCR analysis of changes in the mRNA levels of the proinflammatory genes iNOS, IL-6, TNF-α, and IL-1β. Data are expressed as the mean ± SEM. Data were analyzed using one-way ANOVA. *p < 0.05, **p < 0.01, ***p < 0.001.

To explore the role of TSPO in microglia, we isolated primary microglial cells from WT and *TSPO* ^−/−^ mice and silenced TSPO in BV2 cells by infection with lentivirus containing TSPO shRNA. The purity of isolated primary microglia was checked and is shown in **Figure S1A**. In addition, we found that TSPO was completely deleted in *TSPO* ^−/−^ microglia (**Figure S1B**), and the knockdown efficiency of TSPO in BV2 cells reached 85% (**Figure S1C**). To investigate whether TSPO is involved in microglial M1 state activation, we stimulated primary microglial cells with LPS at four different time points and found that the protein levels of TSPO were significantly increased after LPS stimulation (**Figures 1A, B** **)**, indicating that TSPO might be involved in LPS-induced microglial activation. Moreover, as shown in **Figures 1C–F**, LPS stimulation significantly increased the mRNA levels of proinflammatory markers, including inducible nitric oxide synthase (iNOS), interleukin 1 beta (IL-1β), interleukin 6 (IL-6), and tumor necrosis factor alpha (TNF-α). Knockout (KO) of *TSPO* significantly inhibited the upregulation of proinflammatory factors. Similar phenomena were observed in the TSPO-knockdown stable BV2 cell line (**Figures 1I–L**). Moreover, the protein levels of iNOS were also inhibited in TSPO-knockdown cells upon LPS stimulation (**Figures 1G, H** **)**. Taken together, these results demonstrate that TSPO deficiency inhibits microglial M1 state activation.

### Deficiency of TSPO Inhibits Microglial M2 Polarization

**Figure 2:**
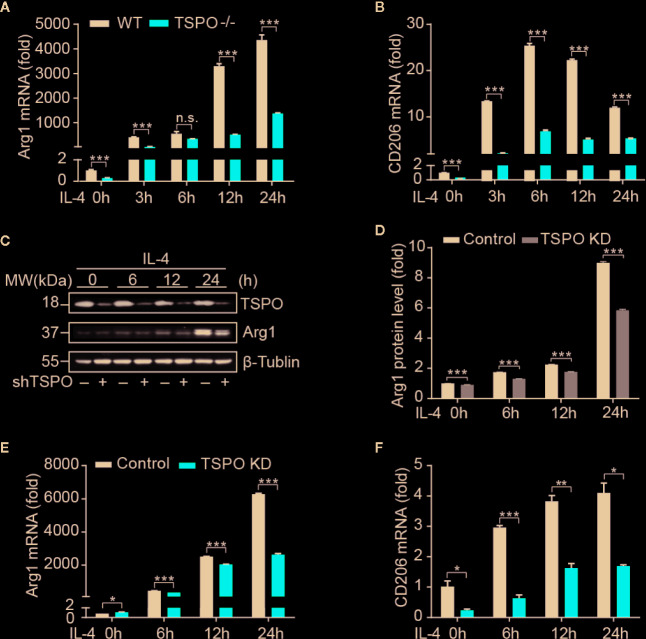
TSPO deficiency inhibits IL-4–induced microglial activation. WT and TSPO-knockout microglial cells were stimulated with IL-4 (1 µg/ml) for the indicated amounts of time. **(A, B)** RT-qPCR determined mRNA changes in the M2 markers Arg1 and CD206. The same conditions were applied to shvector and shTSPO BV2 cell lines. **(C, D)** Western blot analysis of Arg1 protein level changes. **(E, F)** RT-qPCR analysis of mRNA changes in Arg1 and CD206. Data are expressed as the mean ± SEM. Data were analyzed using one-way ANOVA. *p < 0.05, **p < 0.01, ***p < 0.001.

**Figure 3:**
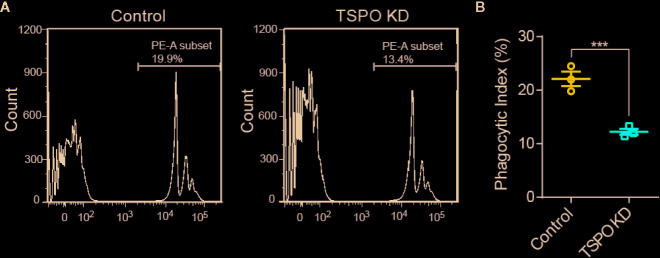
TSPO deficiency impairs phagocytosis in BV2. **(A, B)** Phagocytosis was measured by incubating latex beads in shvector and shTSPO BV2 cells. Data are expressed as the mean ± SEM. Data were analyzed using one-way ANOVA. ***p < 0.001.

In order to investigate the role of TSPO in microglial M2 polarization, IL-4 was used to stimulate WT and *TSPO* KO primary microglial cells at the indicated times. We found that Arg1 and CD206 (M2 markers) were both increased significantly after IL-4 stimulation, while TSPO deficiency dramatically reduced the upregulation of M2 markers (**Figures 2A, B** **)**. Similar trends were shown in the TSPO-knockdown stable BV2 cell line (**Figures 2C–F**), suggesting that TSPO deficiency also inhibits microglial M2 state activation. Phagocytosis is another characteristic of the microglial M2 state. Here, we found that deficiency of TSPO could significantly decrease microglia phagocytosis of fluorescent latex beads detected by flow cytometry (**Figures 3A, B** **)**. Taken together, these data further implied that TSPO plays a vital role in microglial polarization, as TSPO deficiency attenuates the ability of microglial M1 and M2 polarization.

### TSPO Deficiency Dampens Microglial Mitochondrial Function

**Figure 4:**
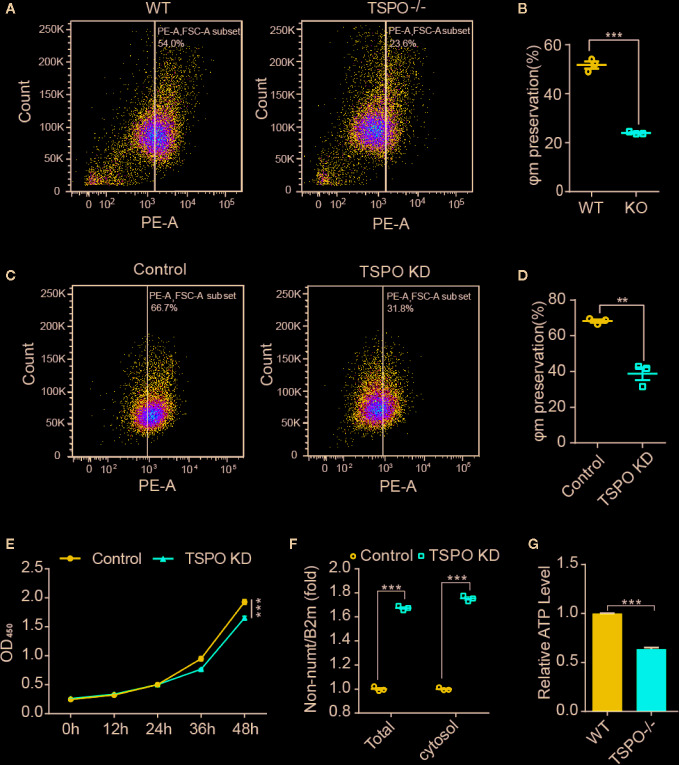
TSPO knockout dampens microglial mitochondrial function. **(A, B)** The mitochondrial membrane potential (Ψm) of WT and TSPO−/− microglial cells was measured by TMRM fluorescence (n=3 biological replicates). **(C, D)** The mitochondrial membrane potential (Ψm) of shvector and shTSPO cells was measured by TMRM fluorescence. **(E)** shvector and shTSPO knockdown cell lines were placed in 96-well plates at the same density, and OD450 values were detected by adding CCK at specific times. **(F)** The relative total mtDNA production and the amount of mtDNA released in the cytosol of WT and TSPO−/− microglia cells were quantified by RT-qPCR with primers specific for a region of mtDNA that is not inserted into nuclear DNA (non-NUMT) and primers specific for nDNA (B2m). **(G)** Total ATP production of WT and TSPO−/− microglial cells. Data were analyzed using one-way ANOVA. **p < 0.01, ***p < 0.001.

As TSPO is located in the mitochondrial outer membrane and TSPO is involved in the functional regulation of mitochondria (Liu et al., 2017), we hypothesize that TSPO might regulate microglial activation by affecting mitochondrial function. To address this, we assessed the effect of TSPO deficiency on the mitochondrial membrane potential (Ψm) of primary microglia and the TSPO-knockdown stable BV2 cell line by TMRM fluorescence. We found that TMRM (a cell-permeant dye that accumulates in active mitochondria with intact membrane potentials) accumulation was markedly reduced in TSPO-deficient cells, as detected by flow cytometry, which was also observed in *TSPO* knockout primary microglial cells (**Figures 4A–D**).

As shown in **Figure 4E**, detected by CCK8 assay, we found that knockdown of TSPO inhibited the proliferation of BV2 cells. In addition, mitochondrial DNA copy number was increased in the TSPO-knockdown groups (**Figure 4F**), with an increase in the amount of mitochondrial DNA released into the cytosol (**Figure 4F**). Furthermore, we found that total ATP production in *TSPO* KO primary microglial cells was largely reduced compared to that in WT cells (**Figure 4G**). Collectively, these findings indicate that TSPO deficiency reduces mitochondrial membrane potential and impairs mitochondrial function.

### TSPO Deficiency Inhibits Energetic Metabolism by Suppressing Mitochondrial OXPHOS and Glycolysis in Microglia

**Figure 5:**
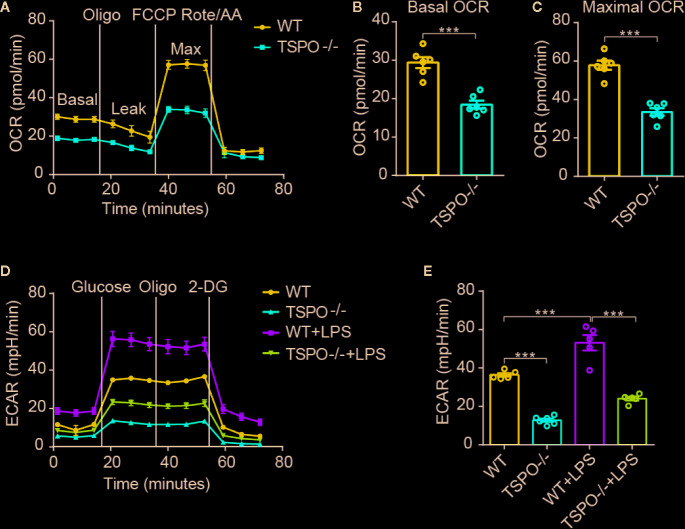
TSPO deficiency restrains mitochondrial OXPHOS and glycolysis. **(A)** OCR measurements of WT and TSPO−/− microglial cells. p-Trifluoromethoxy carbonyl cyanide phenylhydrazone (FCCP) is a reversible inhibitor of OXPHOS; Rote and AA are inhibitors of mitochondrial complex I and complex III, respectively. **(B, C)** Statistics on basic OCR and maximum OCR. **(D, E)** ECAR measurements of WT and TSPO−/− microglial cells in the presence or absence of LPS (1 µg/ml) treatment for 24 hours. The basal ECAR were calculated. Oligomycin (Oligo) is an inhibitor of ATP synthase; 2-deoxyglucose (2-DG) is a glucose analog. Data are expressed as the mean ± SEM. ***p < 0.001; Student’s t-test or one-way ANOVA **(B, C)**, followed by Tukey’s multiple comparison test **(E)**.

As OXPHOS and glycolysis are two main sources of ATP production, we hypothesized that mitochondrial inner membrane damage may result in a decrease in the transmission function of the respiratory electron transport chain, thus affecting the production of ATP. In order to address this possibility, the oxygen consumption rate (OCR), which represents mitochondrial oxidative respiration, was detected. Sequential compounds, including oligomycin FCCP antimycin A & rotenone, were injected to measure basal respiration, ATP production, proton leakage, maximal respiration, spare respiratory capacity, and nonmitochondrial respiration. We found that basal respiration and spare maximal respiration were all significantly decreased in *TSPO* KO microglial cells compared to WT cells (**Figures 5A–C**). Furthermore, we measured the extracellular acidification rate (ECAR), which represents glycolytic function. Interestingly, we found that *TSPO* KO also decreased ECAR in microglia (**Figures 5D, E** **)**. Moreover, the enhanced ECAR driven by LPS stimulation was almost abolished in TSPO deficient microglia. Taken together, these data demonstrate that TSPO is involved in microglial energetic metabolism by maintaining the normal functions of mitochondrial OXPHOS and glycolysis.

## Discussion

Activated microglia play a critical role in nervous system diseases; microglial activation includes two types, namely, inflammatory M1 and neuroprotective M2 (David and Kroner, 2011; Hu et al., 2015). Increasing evidence shows that microglial activation is a potential target to treat a variety of neurological diseases, such as Alzheimer’s disease (AD), Parkinson’s disease (PD), and stroke (Stone et al., 2009; Macrez et al., 2011; Gold and El Khoury, 2015). TSPO acts as a potential biomarker of neuroinflammation, whose ligands are being developed for neuroimaging in the clinic (Rupprecht et al., 2010). However, the mechanism underlying the upregulation of TSPO during neuroinflammation and the role of TSPO in the activation of microglia remain unclear. Here, our findings suggest that TSPO-deficient microglia remain in an improperly homeostatic state instead of responding appropriately to stimulation by LPS and IL-4. Similar trends were observed in TREM2, whose deficiency causes microglial activation disorder and accelerates disease development in a mouse model of AD (Ulland et al., 2017). The accumulation of amyloid-β (Aβ) and misfolded proteins are major neuropathological findings for neurodegenerative diseases. Microglia, as inspectors of the CNS, play critical roles in maintaining brain homeostasis by removing cellular debris and misfolded proteins (Lucin et al., 2013; Diaz-Aparicio et al., 2016). Furthermore, impaired phagocytosis has been shown to aggravate the development of neurodegenerative disease (Lucin et al., 2013). Here, we demonstrate that TSPO deficiency significantly inhibits the phagocytic function of microglia, suggesting that TSPO might be involved in misfolded protein-related neurodegenerative diseases.

The phagocytosis of microglia requires dynamic cytoskeletal reorganization, which requires a large amount of energy (Pan et al., 2019). Previous reports also indicate that phagocytic receptor deficiency is always accompanied by a decrease in energy generation (Ulland et al., 2017). In the present study, we found that TSPO-deficient microglia generate less energy than wild-type microglia. It is well known that mitochondria provide primary energy in the form of ATP. TSPO forms a complex composed of the proteins VDAC and ANT, which reside in the mitochondrial permeability transition pore (MPTP), indicating that TSPO may play an important role in mitochondria (Papadopoulos et al., 2006; Papadopoulos and Lecanu, 2009). Increasing evidence shows that the regulation of mitochondrial function is becoming an attractive therapeutic strategy for CNS diseases involving mitochondrial dysfunction, including neurodegenerative and psychiatric disorders. Our research reveals that TSPO deficiency significantly reduces microglial mitochondrial membrane potential (MMP), which is always accompanied by impairment of mitochondrial function and inhibition of oxidative phosphorylation (OXPHOS), which is consistent with one previous study that *TSPO* knockout inhibits OXPHOS in human microglia C20 cell line (Milenkovic et al., 2019). In addition to OXPHOS occurring in the inner mitochondrial membrane, we showed that glycolysis, another pathway for ATP production in the cytosol, is also inhibited in TSPO-deficient microglia. Damage to OXPHOS and glycolytic also significantly inhibits microglial survival. Our results indicate that TSPO deficiency significantly inhibit microglia proliferation. In addition, increased amount of released mitochondrial DNA (mtDNA) also indicates that TSPO deficiency induces microglia mitochondrial inner membrane damaged. Together, sustaining microglial metabolism is a potential avenue for AD and other neurodegenerative diseases associated with microglial dysfunction.

In the last decade, an increasing number of studies have reported that TSPO ligands are neuroprotective in various experimental pathological conditions, including models of experimental autoimmune encephalomyelitis (EAE), chronic unpredictable stress (CUS), post-traumatic stress disorder (PTSD) and neurodegenerative diseases (Daugherty et al., 2013; Zhang et al., 2014a; Zhang et al., 2014b). However, the exact mechanism behind the therapy is still unclear, as TSPO ligands are not specific and selective enough, the beneficial effects of TSPO ligands in therapy cannot truly explain the TSPO effects (Gut et al., 2015; Bader et al., 2019). Moreover, our study provides a new understanding of microglia activation from the perspective of cellular metabolism. It is worth noting that Bae et al. reported that TSPO deficiency promotes microglial inflammation, which is contrary to our results (Bae et al., 2014; Wang et al., 2014). These conflicting observations may be caused by the differences in LPS concentration for stimulating and LPS stimulation duration. We argue that higher concentration of LPS might induce damage to the microglial energy metabolism and cause microglia into an improperly metabolic state. Together with other laboratory results, a better understanding of TSPO function we demonstrated here is beneficial for the application of TSPO ligands in laboratory experiments and clinical trials.

In summary, we demonstrate that TSPO is highly expressed in reactive microglia, revealing that TSPO plays a critical role in mediating microglial activation. Mechanistically, TSPO deficiency impairs microglial mitochondrial function and weakens cellular energy metabolism, suggesting that TSPO might be a promising treatment target for CNS diseases.

## Data Availability Statement

All datasets generated for this study are included in the article/**Supplementary Material**.

## Ethics Statement

All experimental animal procedures were approved by the Institutional Animal Care and Use Committee of the Beijing Institute of Basic Medical Sciences.

## Author Contributions

RY performed all the experiments and wrote the manuscript. RP and XL provided some help in the molecular and cellular experiments. CS helped to generate the TSPO knockout mice. YL, JC, and JX developed the concepts and designed and supervised the project. All authors contributed to the article and approved the submitted version.

## Funding

This work was supported by the Natural Science Foundation of China (81773708 and 81173036); the Nationa Nature Science Foundation of China (81630026, 81870839); the open fund of the Key Laboratory of Modern Preparation of TCM, Ministry of Education, Jiangxi University of Traditional Chinese Medicine (TCM-201915).

## Conflict of Interest

The authors declare that the research was conducted in the absence of any commercial or financial relationships that could be construed as a potential conflict of interest.
