# Supplementary material for: Translocator Protein 18 kDa (TSPO) Deficiency Inhibits Microglial Activation and Impairs Mitochondrial Function
Source: Front Pharmacol. 2020 Jun 30;11:986. doi: 10.3389/fphar.2020.00986 (PMC7339871; doi:10.3389/fphar.2020.00986)
Supplement: Supplementary file 1 [file Table_1.pdf]

Table 1: The primer list for the genes tested in this study.

Gene Name	Types of primer	Sequence
Mouse β-actin	Forward	5’-GGCTGTATTCCCCTCCATCG-3’
	Reverse	5’-CCAGTTGGTAACAATGCCATGT-3’
Mouse GAPDH	Forward	5’-AGGTCGGTGTGAACGGATTTG-3’
	Reverse	5’-GGGGTCGTTGATGGCAACA-3’
Mouse TSPO	Forward	5’-GCCTACTTTGTACGTGGCGAG-3’
	Reverse	5’-ATGGCTGAATACAGTGTTGCC-3’
Mouse IL-1β	Forward	5’-TGTAATGAAAGACGGCACACC-3’
	Reverse	5’-TCTTCTTTGGGTATTGCTTGG-3’
Mouse IL-6	Forward	5’-GCTACCAAACTGGATATAATCAGGA-3’
	Reverse	5’-CCAGGTAGCTATGGTACTCCAGAA-3’
Mouse iNOS	Forward	5’-GTTCTCAGCCCAACAATACAAGA-3’
	Reverse	5’-GTGGACGGGTCGATGTCAC-3’
Mouse TNF-α	Forward	5’-CAGGCGGTGCCTATGTCTC-3’
	Reverse	5’-CGATCACCCCGAAGTTCAGTAG-3’
Mouse Arg-1	Forward	5’-CTCCAAGCCAAAGTCCTTAGAG-3’
	Reverse	5’-AGGAGCTGTCATTAGGGACATC-3’
Mouse CD206	Forward	5’-CTCTGTTCAGCTATTGGACGC-3’
	Reverse	5’-CGGAATTTCTGGGATTCAGCTTC-3’
Mouse Non-numt	Forward	5′-CTAGAAACCCCGAAACCAAA-3′
	Reverse	5′-CCAGCTATCACCAAGCTCGT-3′
Mouse B2m	Forward	5′-ATGGGAAGCCGAACATACTG-3′
	Reverse	5′-CAGTCTCAGTGGGGGTGAAT-3
