# Supplementary material for: Translocator Protein 18 kDa (TSPO) Deficiency Inhibits Microglial Activation and Impairs Mitochondrial Function
Source: Front Pharmacol. 2020 Jun 30;11:986. doi: 10.3389/fphar.2020.00986 (PMC7339871; doi:10.3389/fphar.2020.00986)

Translocator protein 18 kDa (TSPO) deficiency inhibits microglial activation and impairs mitochondrial function

Running Title: TSPO deficiency inhibits microglial activation

Figure. S1. Detection of TSPO knock out and knock-down efficiency

(A) Representative images of co-staining for the microglia-specific marker Iba1 (shown in red) and nuclei (stained with DAPI, shown in blue). The 4 groups contained a total of n=340 cells. (B) Western blot analysis of the efficiency of TSPO knockout in primary microglia isolated from TSPO-/- mice, with WT mice for comparison. (C) Western blot analysis of TSPO knockdown efficiency after infection with a lentivirus encoding TSPO shRNA versus vector shRNA. Data are expressed as the mean ± SEM. *$p<0.05$, **$p<0.01$, ***$p<0.001$, one-way ANOVA.

Supplementary figure:

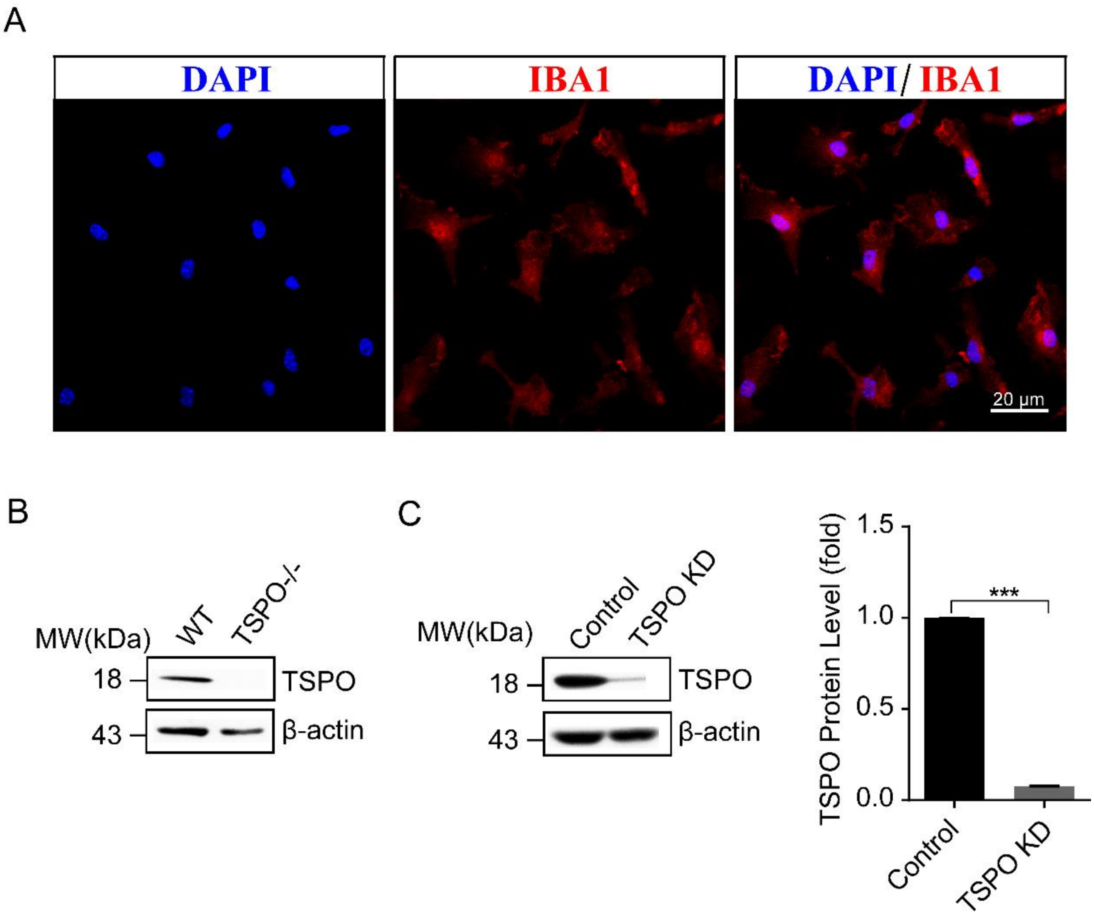

Supplement: Supplementary file 2 [file Image_1.pdf]
